# Preliminary Proof-of-Concept Testing of Novel Antimicrobial Heat-Conducting “Metallic” Coatings Against Biofouling and Biocorrosion

**DOI:** 10.3389/fmicb.2022.899364

**Published:** 2022-06-30

**Authors:** Di Wang, Timothy D. Hall, Tingyue Gu

**Affiliations:** ^1^Shenyang National Lab for Materials Science, Northeastern University, Shenyang, China; ^2^Department of Chemical and Biomolecular Engineering, Institute for Corrosion and Multiphase Technology, Ohio University, Athens, OH, United States; ^3^Faraday Technology, Inc., Englewood, OH, United States

**Keywords:** biofilm, biofouling, biocorrosion, coating, antimicrobial, heat exchanger

## Abstract

NiMo (nickel-molybdenum) and NiMo with embedded CeO_2_ nanoparticles (NPs; 100 nm) were tested as antimicrobial coatings (~15 μm thickness) on titanium (Ti) surfaces using an electrochemical process for heat exchanger applications onboard marine vessels. Preliminary static biofouling and biocorrosion (also known as microbiologically influenced corrosion) assessments were carried out in glass bottles using pure-culture *Desulfovibrio vulgaris*, a sulfate-reducing bacterium (SRB), in deoxygenated ATCC 1249 medium at 37°C, and using an alga (*Chlorella vulgaris*) mixed with general heterotrophic bacteria (GHB) in enriched artificial seawater at 28°C. It was found that the coating containing NiMo/CeO_2_ NPs were much more effective than NiMo in preventing SRB biofilm formation with an efficacy of 99% reduction in *D. vulgaris* sessile cells after 21 day incubation. The coating also exhibited a 50% lower corrosion current density compared to the uncoated Ti against SRB corrosion. Both NiMo and NiMo/CeO_2_ NP coatings achieved 99% reduction in sessile algal cells. Confocal laser scanning microscopy (CLSM) biofilm images indicated a large reduction of sessile GHB cells. The CLSM images also confirmed the biocidal kill effects of the two coatings. Unlike polymer coatings, the “metallic” coatings are heat conductive. Thus, the corrosion resistant antifouling coatings are suitable for heat exchanger applications.

## Introduction

Seawater biofouling is a major threat in heat exchanger operations. It decreases the heat transfer efficiency and service life of heat exchangers (Müller-Steinhagen et al., 2011; García and Trueba, 2019). The formation of deposits caused by biofouling on the heat exchanger metal surfaces increases surface roughness and decreases cross-sectional flow area, which leads to higher friction loss in fluid flow (Kazi, 2012, 2020). Mitigation methods, including surface scrubbing, fluidizing bed heat exchangers, cleaning-in-place and dosing antifouling chemicals, are the main ways to tackle biofouling (Berce et al., 2021). Conventional approaches to treat biofouled heat exchangers by periodic electrochlorination or acid flushes are costly and environmentally hazardous. Huge costs are associated with heat exchanger biofouling losses, but there is still a lack of research to develop heat-conducting antifouling coatings to heat exchangers (Kazi, 2012).

Biofouling and biocorrosion can be caused by unwanted growth of biofilms (Vanysacker et al., 2014; Achinas et al., 2020). Sulfate-reducing bacteria (SRB), which can grow anaerobically beneath an aerobic or facultative biofilm, are the ubiquitous bacteria in biocorrosion (Jia et al., 2019; Rasheed et al., 2019; Wang et al., 2020, 2021b,c). Titanium (Ti) and Ti alloys have good mechanical properties, and high corrosion resistance due to the thin and dense oxidized films on the Ti surface that provide excellent passivation against corrosion (Yuan et al., 2008; Lu et al., 2018; Dias Corpa Tardelli et al., 2020). However, they are susceptible to biofilm attachment, and biocorrosion by SRB and other microbes (Yuan et al., 2008; Khan et al., 2019). Ti heat exchanger tubing biofouling occurs naturally during open water-cooling operations on both military and commercial vessels (Rajagopal et al., 2012; Kazi, 2020). The cooling water side operating temperature range is typically mild, which is suitable for microbial growth (Agree, 1991).

Seawater heat exchangers are plagued by biofouling which results in severe degradation of performances. Furthermore, heat exchanger failure can lead to ships being disabled. For example, USS Zumwalt (DDG class) broke down in the Panama Canal in 2016, presumably due to a heat exchanger failure and seawater leaks. It had to be towed to port (Doubleday, 2016). A protective coating is desired to resist biofouling and biocorrosion caused by microbial biofilms. Polymer coatings are a common approach to combat corrosion (Huang et al., 2012). There are different antibacterial polymer coatings, such as antibacterial coating of agarose polysaccharide (Xu et al., 2017), anti-algal coating of poly(ethylene glycol) and anti-biocorrosion coating of polyethyleneimine-chitosan (Xu et al., 2016), and anti-biofilm coating of polyglycerol (Pranantyo et al., 2016). Antimicrobial agents can be blended into polymer coatings to mitigate biofilm formation. However, polymer coatings do not conduct heat well and their low erosion resistance is also a major problem (Eliaz, 2019). Thus, polymer coatings are used on external pipeline surfaces, but they are not suitable for internal pipeline or heat exchanger applications.

Nickel-molybdenum (NiMo) “metallic” coatings show excellent corrosion resistance in harsh environments, such as acidic, high temperature, high wear environments (Xu et al., 2019; Zamani Meymian et al., 2020). Because of their superior hardness (1,000 Knoop), good thermal conductivity of 11.1 W/(mK) vs. 16.0 W/(mK) for Ti at 20°C (Gale and Totemeier, 2004), and high corrosion resistance in seawater environments, NiMo is a good choice as a metallic base coating material. The presence of Mo in 316 stainless steel (SS) is known to inhibit the growth of SRB biofilms (Wang et al., 2021a).

During heat exchanger operations in the field, mixed-culture biofilms form on tubing surfaces. The top layer of a biofilm is aerobic, but bottom tends to be anaerobic. A coating’s resistance to SRB is important because SRB sessile cells live in the anaerobic niche directly on the base metal surface underneath the top biofilm (Radhika et al., 2006). SRB cells produce highly reactive sulfide species that can react with biocidal metal ions (Enning and Garrelfs, 2014). SRB are known to resist copper ion toxicity because their metabolic product H_2_S can precipitate biocidal Cu^+^ and Cu^2+^ extremely well. Copper ion toxicity only slows down SRB growth slight at the beginning (Dou et al., 2018; Wang et al., 2020). Cu-bearing SS is very effective against *Pseudomonas aeruginosa* biofilm, but it has no efficacy against SRB biofilm formation (Lou et al., 2016; Liu et al., 2018). Robust SRB biofilms can grow on Cu surfaces (Dou et al., 2018).

It was recently shown that when cerium element (in the form of CeO_2_) was over 0.18 wt.% in Cu-bearing 2205 duplex stainless steel (DSS), SRB biofilm formation was inhibited effectively (Yin et al., 2021). It showed superior SRB resistance compared with 2205 DSS and 2205 Cu-DSS without Ce. Nanoscale CeO_2_ has the excellent antibacterial effects in biology and medical sciences comparing with other metal oxides (Qi et al., 2020).

In this preliminary work, NiMo coatings with varying concentrations of biocidal CeO_2_ nanoparticle (NP) inclusions were applied to grade 2 Ti substrate. The antifouling efficacies of the coatings were evaluated using sessile cell counts and confocal laser scanning microscopy (CLSM) images after 21 day incubation in ATCC 1249 culture medium inoculated with *Desulfovibrio vulgaris*, and 21-day incubation in enriched artificial seawater (EASW) inoculated with *Chlorella vulgaris* (a common algal species) mixed with general heterotrophic bacteria (GHB). Two best performing coatings were chosen for further evaluation using electrochemical tests for SRB corrosion resistance. An empirical biofouling resistance correlation was established using the new concept of biofouling resistance equivalent number (BREN).

### Experimental

Table 1 shows the test matrix. Various experimental detailed are described in the following sub-sections.

**Table 1:** Test matrix for SRB and alga.

Microorganism	*D. vulgaris* (pure culture)	*C. vulgaris* (mixed with GHB)
Culture medium	ATCC 1249 medium	EASW
Coupon	Ti coupons with and without antibiofilm coatings	Ti coupons with and without antibiofilm coatings
Culture medium volume	50 ml in 125 ml anaerobic vials	200 ml in 450 ml glass bottles
Temperature	37°C (optimal for growth)	28°C
Incubation Time	21 days	21 days
Analysis	Cell counting and CLSM imaging	Cell counting and CLSM imaging

### Preparation of NiMo-Biocidal Coatings

**Figure 1:**
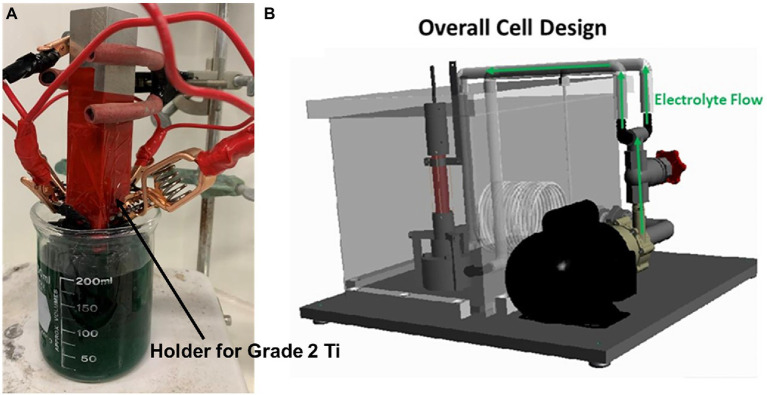
Setup for a rectangular Ti sample used for preliminary coating screening **(A)**, and tool for applying a coating to Ti surface **(B)**.

The Biocidal CeO_2_ nanoparticle were purchased as suspension in water from US Research Nanomaterials, Inc. (Houston, TX, United States). The CeO_2_ NPs (100 nm) were incorporated within a metal (NiMo) matrix on Ti surface by electrocodeposition. The advantage of fabricating composites in this way is the ability to produce materials with very uniform particle dispersion within the metal matrix, owing to particle suspension in an aqueous solution that reduces their agglomeration (Sautter, 1963). Pulsed current (PC) was applied in the electrocodeposition process. In the PC scheme, the applied current was interrupted by an off-time, t_off_, where typically open circuit potential (OCP) conditions were maintained. The PC scheme promoted a nanocrystalline structure in the NiMo, and CeO_2_ NP inclusions that were less agglomerated in comparison to those observed under direct current (DC) mode. In this work, DC, high frequency PC and low frequency PC were used to produce different coatings. Higher frequency has the potential to increase the amount of CeO_2_ included into the coating. Electrochlorination process could have less cost if low frequency used. The FARADAYIC antifouling electrocodeposition process was utilized to apply NiMo-biocidal coatings directly to grade 2 Ti substrate (Yan et al., 2018). Figure 1 shows the setup for the whole process. A NiMo electrodeposition solution that consisted of 0.02 M Mo (from sodium molybdate), 0.2 M Ni (from nickel sulfate), and 0.18 M tri-sodium citrate was used. This solution’s pH was increased to 9.5 with ammonium hydroxide prior to each trial. CeO_2_ NPs at 4 wt.% were added to the NiMo electrodeposition solution at pH 9.5 to create Ce-NiMo coatings on Ti. The coating did not significant change the wrought Ti surface roughness (3 μm). In order to achieve a consistent surface from which to initiate the electrodeposition procedure a grit blasting routine with 40 to 80 μm AlO_3_ powder was carried out. This pretreatment left a uniform surface with 3 μm roughness on which the coating was applied. Coated and uncoated coupons were sanitized using pure isopropanol and dried under UV light before they were immersed in EASW for incubation.

### Bacterium, Alga, Culture Media, and Chemicals

*D. vulgaris* (ATCC 7757 strain), a commonly used SRB species for biocorrosion studies, was incubated anaerobically in 125 ml vials with 50 ml deoxygenated ATCC 1249 culture medium at 37°C for 21 days. The composition of ATCC 1249 medium was (g/L): MgSO_4_·7H_2_O 4.1, sodium citrate·2H_2_O 5.7, CaSO_4_·2H_2_O 1.0, NH_4_Cl 1.0, K_2_HPO_4_, 0.5, sodium lactate 3.5, yeast extract 1.0, Fe(NH_4_)_2_(SO_4_)_2_·6H_2_O 1.38. *C. vulgaris* mixed with GHB was incubated aerobically in 200 ml EASW in 450 ml glass vessels at 28°C for 21 days. A lower temperature was used for better *C. vulgaris* growth. The abundant GHB in the mixed culture helped validate coating efficacy against biofouling bacteria other than *C. vulgaris* eukaryotic algae. The EASW composition was (g/L): Na_2_SO_4_ 3.91, NaCl 23.476, NaHCO_3_ 0.192, KBr 0.096, KCl 0.664, H_3_BO_3_ 0.026, SrCl_2_·6H_2_O 0.040, MgCl_2_·6H_2_O 10.610, CaCl_2_·2H_2_O 1.469, yeast extract 1.0, tri-sodium citrate 0.5, sodium lactate 3.5, CaSO_4_·0.5H_2_O 0.1, NH_4_Cl 0.1, MgSO_4_·7H_2_O 0.71, Fe(NH_4_)_2_(SO_4_)_2_·6H_2_O 1.38, K_2_HPO_4_ 0.05.

The initial pH values of the culture media were neutralized to 7.0 by adding a 5% (w/w) HCl solution or 5% (w/w) NaOH solution for both cultures. The culture media and lab tools such as tweezers were sterilized in an autoclave for 20 min at 121°C. After autoclaving, the ATCC 1249 medium was sparged with filter-sterilized N_2_ to deoxygenate for at least 1 h. A concentrated L-cysteine stock solution was used to add 100 ppm (w/w) L-cysteine (final concentration) to the ATCC 1249 culture medium after filter-sterilized N_2_ sparging as an oxygen scavenger to reduce dissolved oxygen further. Following EASW sterilization, deoxygenation was not needed for EASW used for *C. vulgaris* incubation because *C. vulgaris* growth was aerobic.

### Cell Counting and CLSM Imaging

After incubation, the immersed coupons were retrieved from the culture medium. SRB sessile cell counts were enumerated using a hemocytometer. For the SRB sessile cell enumeration, the SRB biofilm covered coupons were first rinsed in pH 7.4 phosphate buffered saline (PBS) solution 3 times. The PBS solution was sterilized for 20 min and sparged with filtered-sterilized N_2_ for 45 min before use. The biofilm on each coupon surface was scraped off using a sterile brush into a 10 ml PBS solution. The cell suspension was counted under a hemocytometer. Although very small, *D. vulgaris* cells appeared as motile dots which can be distinguished from iron sulfide precipitate particles under a 400X light microscope. This more precise counting method yielded cell counts that were consistent with MPN (most probably number) results (Wang et al., 2020). Eukaryotic *C. vulgaris* cells were seen as green dots under the 400X light microscope (Supplementary Figure S1). Unlike pure-culture *D. vulgaris* cells that are motile, mixed-culture GHB cells in the algal culture could not be counted on a hemocytometer.

CLSM was used to visualize live and dead *D. vulgaris* cells in the SRB biofilms on replicate coupon surfaces (Chen et al., 2015, 2021) and the GHB cells in the algal-GHB mixed-culture biofilms. Each biofilm covered coupon was rinsed with a pH 7.4 PBS buffer to remove the culture medium and planktonic cells before staining. The dyes used to stain the biofilms were the Live/Dead BacLight^™^ Bacterial Viability Kit L7012 (Life Technologies, Grand Island, NY). When observed under CLSM, live bacterial cells showed up as green dots and dead cells red dots. Please note that biofilms on metals are usually non-uniform. Thus, CLSM images should be used qualitatively to show biofilm coverage. The primary purpose of CLSM images in this work was to show the sessile cell kill effect (vs. dispersal effect), which is not achieved by non-biocidal surface properties such as surface roughness or hydrophobicity.

### Electrochemical Measurements

After the cell counting and CLSM imaging results were analyzed for antibiofouling efficacies, the two best performing coatings were chosen to make working electrodes for electrochemical measurements in SRB corrosion. Wrought Ti was used as the control working electrode. In the electrochemical tests, a 3 ml *D. vulgaris* seed culture was used to inoculate 300 ml ATCC 1249 medium in a 450 ml anaerobic glass cell for a 3-d anaerobic incubation at 37°C. A platinum sheet served as the counter electrode. The reference electrode was a saturated calomel electrode (SCE). LPR (linear polarization resistance) was scanned daily from −10 and + 10 mV vs. OCP at 0.167 mV/s. PDP (potentiodynamic polarization) was performed with a scanning rate of 0.167 mV/s at the end of the 3-day incubation period using a voltage range of ±200 mV vs. OCP. Corrosion current densities (*i*_corr_), corrosion potentials (*E*_corr_), and Tafel slopes (*β*_a_ and *β*_c_) were obtained from Tafel analysis of the PDP curves.

### Biofouling Inhibition Modeling

The pitting resistance equivalent numbers (PREN) is often used to reflect the pitting resistance of stainless steel based on the composition of alloying elements. A common empirical correlation to calculate PREN is shown below. It derives from the experimental observation that certain metal elements in stainless steel contribute to its pitting resistance (Kang and Lee, 2013; Rajesh Kannan et al., 2020).


(1) $$\mathrm{PREN}=1.0\times \mathrm{Cr}\%\left(w/w\right)+3.3\times \mathrm{Mo}\%+16\times N\%$$

Inspired by PREN, in this work, a biofouling inhibition model was developed by considering the composition of biocidal metal elements in a coating. A new concept of biofouling resistance equivalent numbers (BREN) was created in this work. The BREN equation, and the BREN number for the SRB and the alga were calculated based on the sessile cell count reduction for different coating compositions.

## Results and Discussion

### Cross-Sectional Images of NiMo Coatings

**Figure 2:**
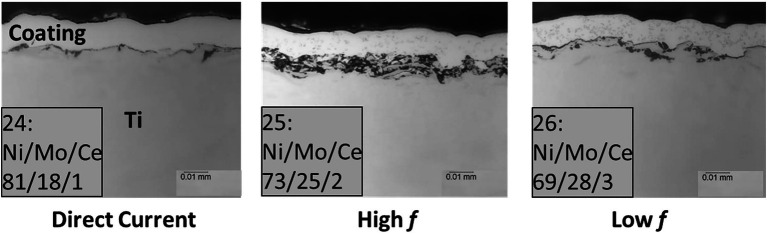
Cross-sectional images of coatings with 100 nm CeO_2_ NP inclusions directly applied to Ti surfaces under various electrocodeposition conditions (DC, high frequency and low frequency). Sample numbers and metal percentages (*w*/*w*) are shown. The top layer above a coating is Epoxy.

The cross-sectional SEM (scanning electron micrography) images (from Metallurgical Solutions Inc., Middletown, OH, United States) in Figure 2 show the NiMo coatings with 100 nm CeO_2_ NP inclusions directly applied to Ti surfaces under various electrocodeposition conditions, in which Mo composition ranged from 5 to 30% (w/w), and CeO_2_ NP composition 0 to 5%. Higher frequency had the ability to increase the amount of CeO_2_ in the coating, while smaller particle sized (50 nm CeO_2_) had the ability to increase the amount of CeO_2_ in the coating. An electrolyte containing 4% (w/w) CeO_2_ was used for electrocodeposition to create the coatings. The elemental composition numbers in Figure 2 indicate mass percentages of each metal element in the coating. Table 2 lists the compositions of Ni/Mo/Ce in different coatings. The biocidal inclusion density of 100 nm CeO_2_ NPs was controlled in the electrocodeposition process.

**Table 2:** Compositions of NiMo and NiMo with CeO_2_ NP inclusion coatings.

Sample [deposition method]	Ni/Mo/Ce (wt%)
NiMo [DC]	81/19/0
NiMo [low *f*]	71/28/0
NiMo [high *f*]	69/31/0
100 nm Ce-NiMo [DC]	81/18/1
100 nm Ce-NiMo [low *f*]	69/28/3
100 nm Ce-NiMo [high *f*]	73/25/2

### Sessile Cell Counts

**Figure 3:**
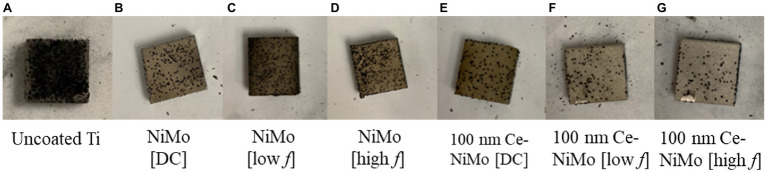
Uncoated Ti coupon (1 cm^2^; **A**), Ti coupon surfaces with NiMo coatings made with different conditions **(B)** NiMo [DC], **(C)** NiMo [low *f*], and **(D)** NiMo [high *f*], Ti coupon surfaces coated with 100 nm Ce in NiMo coatings **(D)** 100 nm Ce-NiMo [DC], and **(E)** 100 nm Ce-NiMo [low *f*], and **(F)** 100 nm Ce-NiMo [high *f*] **(G)** after 21-day SRB incubation at 37°C.

Coupon surfaces with uncoated Ti, with NiMo coatings and 100 nm Ce-NiMo coatings are presented in Figure 3. It shows that the uncoated Ti had far more dark spots sticking to the surface than coated coupons after rinsing with the PBS solution. 100 nm Ce-NiMo coated coupons were cleaner (i.e., less biofouling) than NiMo coated coupons without biocidal CeO_2_ particles. Table 3 shows that, after 21 days of incubation, a healthy SRB biofilm was grown on the wrought Ti surface with a sessile cell count of 1.3 × 10^7^ cells/cm^2^. With NiMo coatings on Ti surfaces, the sessile cell counts showed ~70% reduction. The biofilm thickness (measured using CLSM 3D mode) decreased from 50 μm to less than 30 μm. The 100 nm Ce in NiMo coatings had the best reduction: 100 nm Ce [low *f*] and 100 nm Ce [DC] both achieved 99% (2-log) SRB sessile cell reduction. The biofilm thickness decreased to less than 20 μm. Thus, 100 nm Ce in the NiMo coating improved SRB biofilm prevention considerably. The low *f* (frequency) and DC methods led to slightly better reductions than using a high *f*.

**Table 3:** Sessile cell counts, biofilm thickness from CLSM and final pH after 21-day SRB incubation.

Coating	Sessile cell count (cells/cm^2^)	Reduction	Biofilm thickness (μm)	Final pH
Wrought Ti	1.3 × 10^7^	(control)	50	6.66
NiMo [low *f*]	3.5 × 10^6^	0.57-log	27	6.63
NiMo [DC]	3.0 × 10^6^	0.64-log	29	
NiMo [high *f*]	4.5 × 10^6^	0.46-log	24	
100 nm Ce-NiMo [low *f*]	1.0 × 10^5^	2.1-log	15	6.81
100 nm Ce-NiMo [DC]	1.0 × 10^5^	2.1-log	19	
100 nm Ce-NiMo [high *f*]	7.5 × 10^5^	1.2-log	18	

Table 4 presents the algal sessile cell count and biofilm thickness on three different Ti coupons after 21 days of incubation of a mixed-culture *C. vulgaris* and GHB. On the wrought Ti surface, *C. vulgaris* grew very well, reaching a large sessile cell count of 9.5 × 10^8^ cells/cm^2^. On the NiMo coating and 100 nm Ce-NiMo coating, the algal sessile cell counts decreased by 2-log. The biofilm thickness decreased from 88 μm (no coating) to 31 μm on NiMo coating, and to 29 μm on 100 nM Ce-NiMo coating.

**Table 4:** Algal sessile cell counts after 21-d aerobic incubation.

Coating	Sessile cell count (cells/cm^2^)	Reduction	Biofilm thickness (μm)
Wrought Ti	9.5 × 10^8^	(control)	88
NiMo [DC]	1.2 × 10^7^	1.9-log	31
100 nm Ce-NiMo [DC]	1.5 × 10^7^	1.8-log	29

### CLSM Biofilm Images

**Figure 4:**
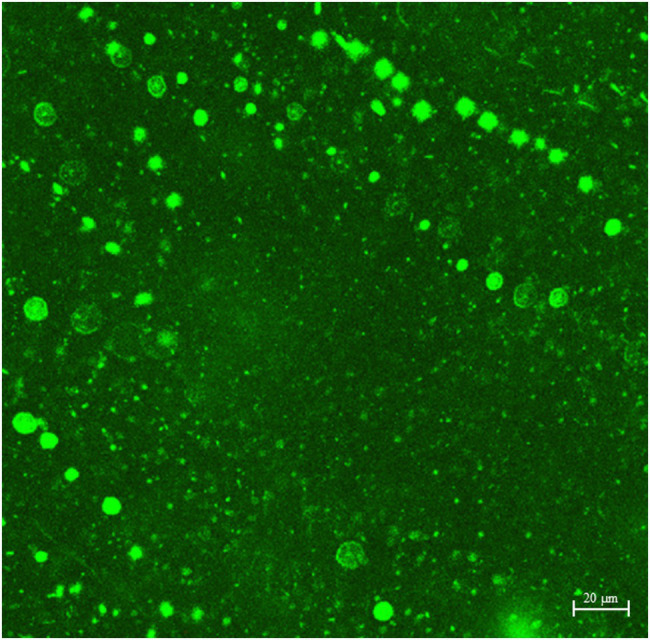
CLSM biofilm image on wrought Ti coupon after 21-day SRB incubation at 37°C.

**Figure 5:**
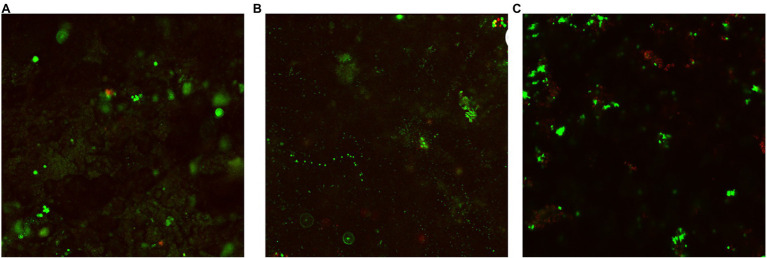
CLSM biofilm images on NiMo coatings with different electrocodeposition conditions after 21-day SRB incubation at 37°C: **(A)** NiMo [DC], **(B)** NiMo [low *f*], and **(C)** NiMo [high *f*].

**Figure 6:**
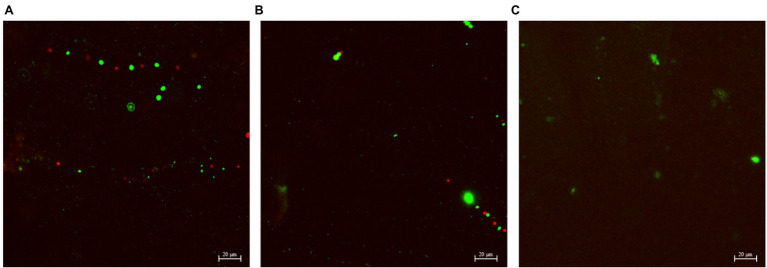
CLSM biofilm images on 100 nm Ce-NiMo coatings with different electrocodeposition conditions after 21-day SRB incubation at 37°C: **(A)** [DC], **(B)** [low *f*], and **(C)** [high *f*].

Figures 4–6 show the CLSM biofilm images on wrought Ti, NiMo coatings and 100 nm Ce-NiMo coating for three different electrocodeposition methods after 21 days of incubation. Figure 4 shows that the wrought Ti surface had a very healthy SRB biofilm coverage with no dead cells (no red spots). On the Ti surface with a NiMo coating, lots of sessile cells were absent, but a few sessile cells still remained on the coating surface (Figure 5). Figure 6 shows that 100 nm Ce-NiMo coating surface had the fewest sessile cells, which is consistent with the SRB sessile cell counts in Table 3. On the coated coupons, some red dots are seen in Figures 5, 6, indicating that the NiMo and Ce-NiMo coatings exhibited kill effect.

**Figure 7:**
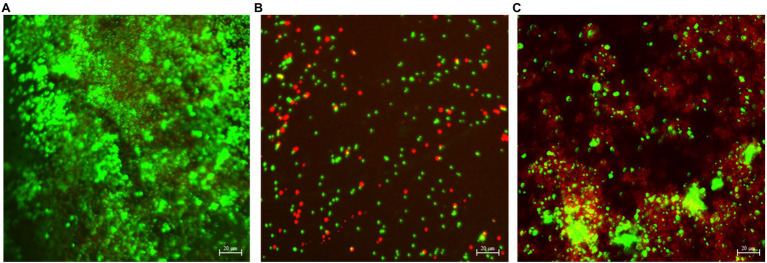
CLSM biofilm images showing GHB cells (green dots for cells and red dead) on wrought uncoated Ti surface **(A)**, NiMo [DC] coating **(B)**, and 100 nm Ce-NiMo [DC] coating **(C)** after 21-day incubation at 28°C.

As shown in Figure 7A, a dense bacterial biofilm grew on the wrought surface. In Figures 7B,C, NiMo coating and 100 nm Ce-NiMo coating surfaces exhibit excellent kill effect of GHB cells with lots of red dots for dead cells, much more than in the CLSM images for SRB biofilms on coated Ti surfaces in Figures 5 and 6. This observation suggests that SRB sessile cells were more recalcitrant than GHB sessile cells. All the CLSM images proved that the efficacy of these antimicrobial coatings against both SRB and GHB cells was through sessile cell kill rather than sessile cell dispersal. Note that the BacLight stain for CLSM was not designed to stain eukaryotic *C. vulgaris* cells in the mixed culture.

### Electrochemical Results

**Figure 8:**
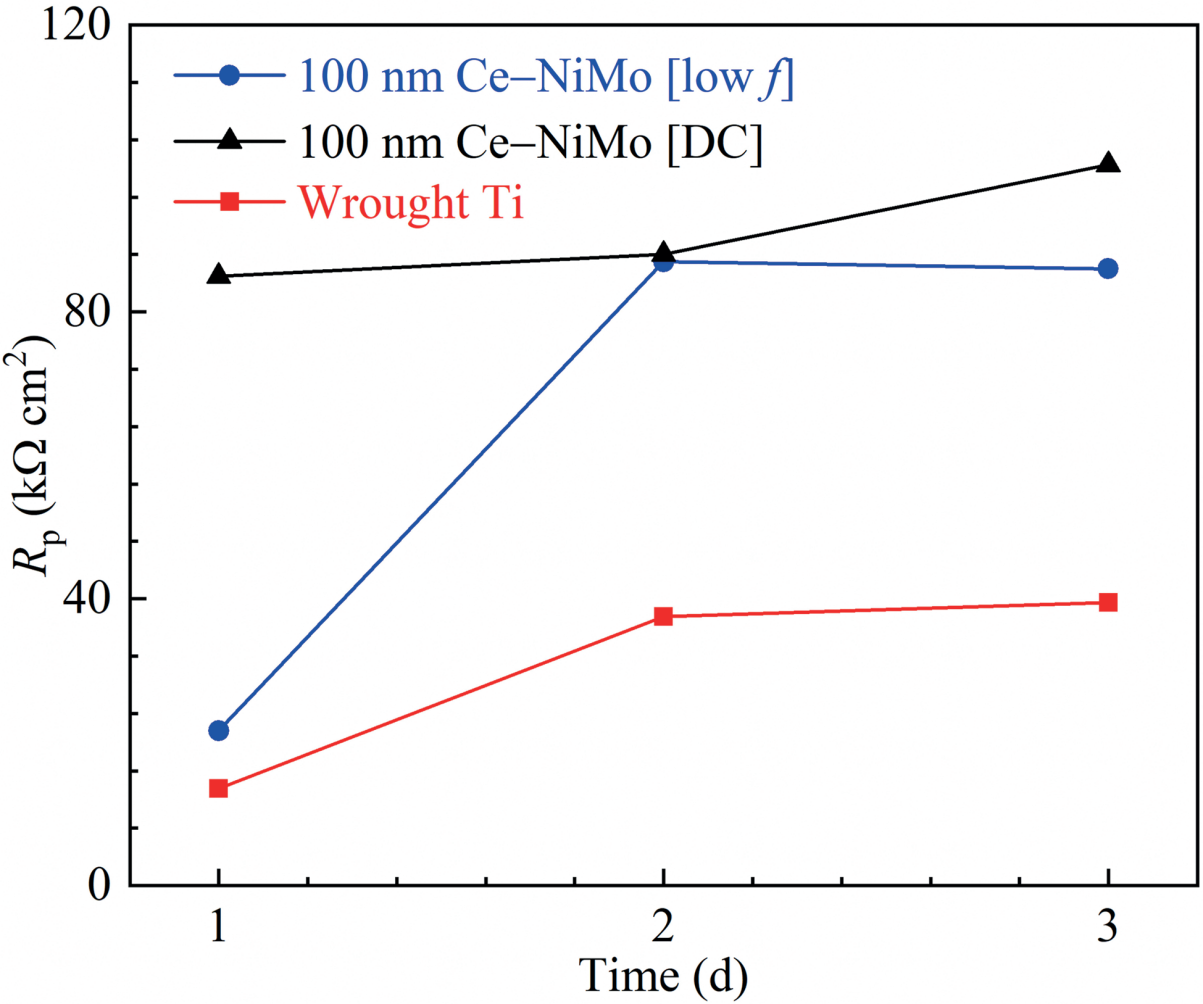
Variations of *R*_p_ vs. time during 3-day SRB incubation in 450 ml glass cells.

Based on the sessile cell counts and biofilm images, 100 nm Ce-NiMo [low *f*] and 100 nm Ce-NiMo [DC] coatings were the two best performing coatings. They were used as working electrodes in electrochemical measurements to evaluate the anti-biocorrosion effects against corrosive *D. vulgaris* biofilm. Biocorrosion tests were not performed for the far less corrosive algal and GHB mixed-culture biofilm. Figure 8 shows the polarization resistance (*R*_p_) for the two coatings and the (uncoated) wrought Ti (control). In theory, a smaller *R*_p_ value means a larger corrosion rate. The wrought Ti had the lowest *R*_p_ in the 3-day incubation, which means it had the highest corrosion rate without coating protection. In comparison, 100 nm Ce-NiMo [DC] had the highest *R*_p_ during the entire 3-day period. The anti-biocorrosion effects of 100 nm Ce-NiMo [low *f*] may had a delay because of the small *R*_p_ in the 1st day.

**Figure 9:**
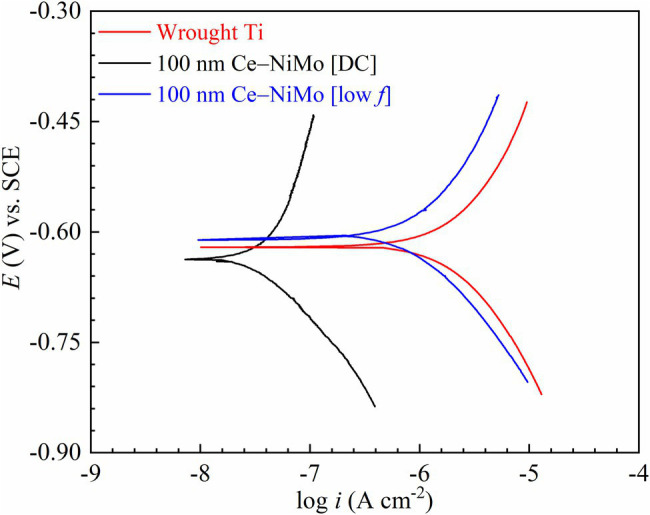
PDP scans in electrochemical glass cells at the end of 3-day SRB incubation.

Figure 9 shows the PDP curves of the three Ti samples in electrochemical glass cells at the end of the 3-day SRB incubation. They were scanned at the end of the incubation per standard practice because the wide scan voltage range might alter the working electrode’s surface. The fitted polarization parameters are shown in Table 5. The (uncoated) wrought Ti coupon had the highest *i*_corr_ value (46.2 nA/cm^2^) compared with the two best performing coatings in the biofouling assessment. 100 nm Ce-NiMo [DC] had the lowest *i*_corr_ value (19.8 nA/cm^2^) with a 57% reduction compared with wrought Ti. Meanwhile, 100 nm Ce-NiMo [low *f*] achieved an *i*_corr_ of 26.5 nA/cm^2^, reflecting a 43% reduction compared with the wrought Ti. The *i*_corr_ trend here is consistent with 1/*R*_p_ trend from LPR, both indicating 100 nm Ce-NiMo [DC] as more corrosion resistant. Corrosion resistant Ti had a very low abiotic corrosion current density (20 nA/cm^2^). The biotic control *i*_corr_ for uncoated wrought Ti was 46.2 nA/cm^2^, indicating the presence of SRB MIC. This value is nonetheless still quite low compared to *D. vulgaris* MIC of carbon steel, which yields 10^3^ higher *i*_corr_ (Jia et al., 2019). In this work, the significant *i*_corr_ reductions on Ti that is already rather resistant to SRB MIC were noteworthy.

**Table 5:** Tafel parameters derived from PDP curves in Figure 9.

Sample	*β*_a_ (mV/dec)	*β*_c_ (mV/dec)	*E*_corr_ (mV) vs. SCE	*i*_corr_ (nA/cm^2^)	*i*_corr_ reduction (%)
Wrought Ti (control)	34	99	−615	46.2	(control)
100 nm Ce-NiMo [DC]	93	148	−628	19.8	57
100 nm Ce-NiMo [low *f*]	33	89	−605	26.5	43

### BREN Parameters

In general, PREN>40 indicates suitability for seawater service and 32 for probable pitting resistance in seawater service for stainless steel (Campbell, 2011). Inspired by the concept of PREN, the following BREN model was proposed,


(2) $$\mathrm{BREN}=a_{1}\times \mathrm{Ni}\%+a_{2}\times \mathrm{Mo}\%+a_{3}\times \mathrm{Ce}\%$$

BREN > 40 is set to suggest excellent (2-log or 99%) reduction of sessile cell count, and 20 for moderate (~70%) reduction of sessile cell count, compared to no coating (or no biocide treatment). Multiple linear regression of SRB sessile cell count reduction data in this work and more suggested that Ni was far more effective than Mo (coefficient ratio 0.95/0.05 or roughly 20:1). Ce was very effective at 1% and further increasing Ce% did not improve efficacy. Thus, the following BREN model correlation for SRB biofouling was established,


(3) $$\begin{array}{l} \mathrm{SRB}\;\mathrm{BREN}=0.\mathrm{3Ni}\%+0.01\mathrm{5Mo}\%\\ +20\;\left(\mathrm{for}\;1\%{\mathrm{CeO}}_{2}\;\mathrm{NPs}\;\mathrm{or higher}\right) \end{array}$$

For *C. vulgaris*, NiMo coating without CeO_2_ NPs was sufficiently effective as shown in the following BREN model correlation,


(4) Algal BREN=0.42Ni%+0.42Mo%

## Conclusion

In this preliminary work, it was found that NiMo coatings on Ti surfaces were effective against *D. vulgaris* biofilm and a mixed-culture biofilm containing *C. vulgaris* and GHB. 100 nm CeO_2_ NPs in NiMo further enhanced the NiMo coating’s efficacy, achieving 2-log reductions in both *D. vulgaris* and *C. vulgaris* sessile cell counts after 21 days of incubation. CLSM biofilm images showed that the GHB sessile cell count was greatly reduced with kill effect. BREN model correlations for *D. vulgaris* and *C. vulgaris* were obtained. More rigorous experimental work with statistical analysis will be needed to evaluate the “metallic” coatings for anti-biofouling and anti-corrosion applications. These coatings are particularly useful when a high thermal conductivity is needed such as in heat exchangers. More microbial species including field microbial consortia need to be tested in future work.

## Data Availability Statement

The raw data supporting the conclusions of this article will be made available by the authors, without undue reservation.

## Author Contributions

TH and TG conceived and designed the experiments. TH fabricated the coatings. DW performed the anti-biofouling and anti-biocorrosion experiments. DW and TG analyzed the data and wrote the manuscript. All authors contributed to the article and approved the submitted version.

## Funding

This work was supported by the U.S. Naval Research Laboratory (Contract #: N68335-21-C-0088).

## Conflict of Interest

TH was employed by the company Faraday Technology, Inc.

The remaining authors declare that the research was conducted in the absence of any commercial or financial relationships that could be construed as a potential conflict of interest.

## Publisher’s Note

All claims expressed in this article are solely those of the authors and do not necessarily represent those of their affiliated organizations, or those of the publisher, the editors and the reviewers. Any product that may be evaluated in this article, or claim that may be made by its manufacturer, is not guaranteed or endorsed by the publisher.
