# Supplementary material for: Preliminary Proof-of-Concept Testing of Novel Antimicrobial Heat-Conducting “Metallic” Coatings Against Biofouling and Biocorrosion
Source: Front Microbiol. 2022 Jun 30;13:899364. doi: 10.3389/fmicb.2022.899364 (PMC9279579; doi:10.3389/fmicb.2022.899364)
Supplement: Supplementary file 1 [file Data_Sheet_1.DOCX]

**Preliminary proof-of-concept testing of novel antimicrobial heat-conducting “metallic” coatings against biofouling and biocorrosion**

Di Wang^1, 2^, Timothy D. Hall^3^, Tingyue Gu^2,*^

^1^Shenyang National Lab for Materials Science, Northeastern University, Shenyang 110819, China.

^2^Department of Chemical and Biomolecular Engineering, Institute for Corrosion and Multiphase Technology, Ohio University, Athens, OH 45701, USA.

^3^Faraday Technology, Inc., Englewood, OH 45315, USA.

^*^Corresponding author: E-mail address: [gu@ohio.edu](mailto:gu@ohio.edu) (T. Gu)


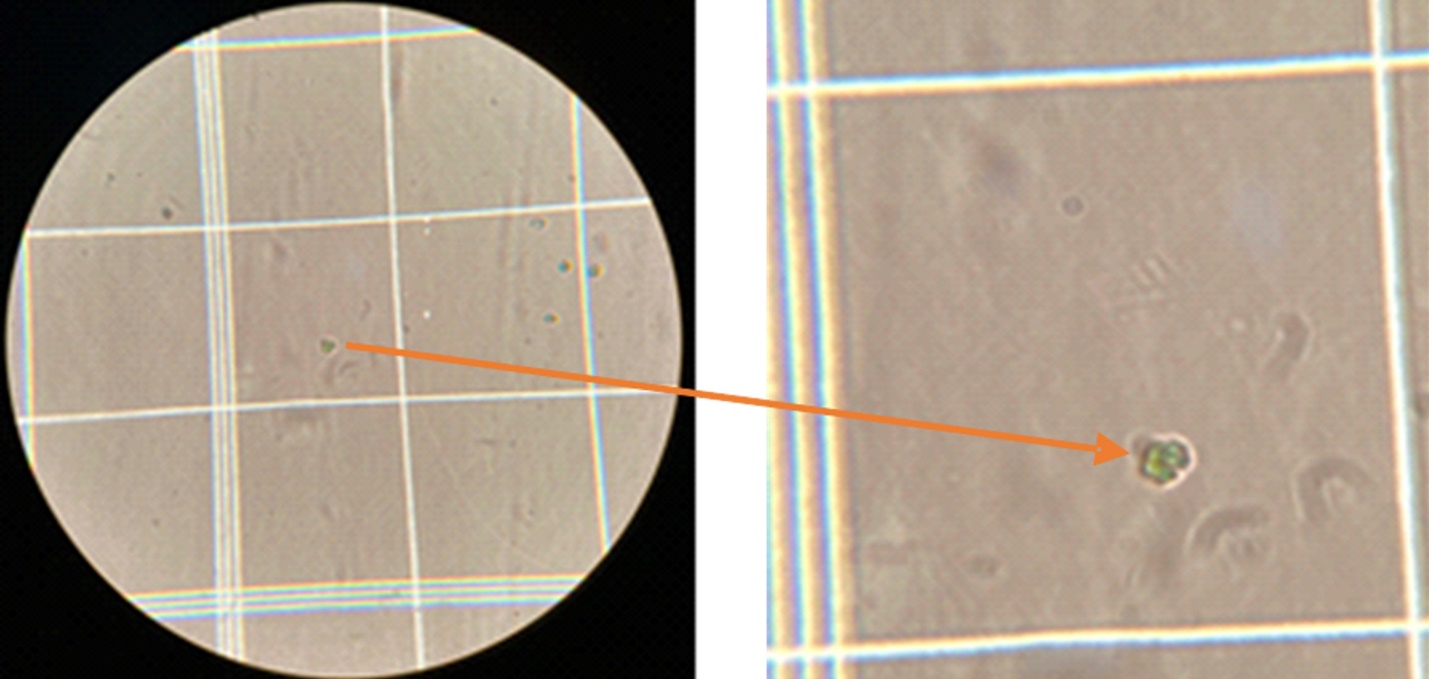


Figure S1. *Chlorella vulgaris* eukaryotic algal cells showing unique green color (each smallest hemocytometer square 0.05 mm × 0.05 mm).
